# Genome-Wide Transcriptional Reorganization Associated with Senescence-to-Immortality Switch during Human Hepatocellular Carcinogenesis

**DOI:** 10.1371/journal.pone.0064016

**Published:** 2013-05-15

**Authors:** Gokhan Yildiz, Ayca Arslan-Ergul, Sevgi Bagislar, Ozlen Konu, Haluk Yuzugullu, Ozge Gursoy-Yuzugullu, Nuri Ozturk, Cigdem Ozen, Hilal Ozdag, Esra Erdal, Sedat Karademir, Ozgul Sagol, Dilsa Mizrak, Hakan Bozkaya, Hakki Gokhan Ilk, Ozlem Ilk, Biter Bilen, Rengul Cetin-Atalay, Nejat Akar, Mehmet Ozturk

**Affiliations:** 1 BilGen Genetics and Biotechnology Center, Department of Molecular Biology and Genetics, Bilkent University, Ankara, Turkey; 2 INSERM - Université Joseph Fourrier, CRI U823, Grenoble, France; 3 Biotechnology Institute, Ankara University, Ankara, Turkey; 4 Department of Medical Biology, Dokuz Eylul University Medical School, Izmir, Turkey; 5 Department of Surgery, Dokuz Eylul University Medical School, Izmir, Turkey; 6 Department of Pathology, Dokuz Eylul University Medical School, Izmir, Turkey; 7 Department of Gastroenterology, Ankara University, Ankara, Turkey; 8 Department of Electronic Engineering, Ankara University, Ankara, Turkey; 9 Department of Statistics, Middle East Technical University, Ankara, Turkey; Inserm, U1052, UMR 5286, France

## Abstract

Senescence is a permanent proliferation arrest in response to cell stress such as DNA damage. It contributes strongly to tissue aging and serves as a major barrier against tumor development. Most tumor cells are believed to bypass the senescence barrier (become “immortal”) by inactivating growth control genes such as *TP53* and *CDKN2A.* They also reactivate telomerase reverse transcriptase. Senescence-to-immortality transition is accompanied by major phenotypic and biochemical changes mediated by genome-wide transcriptional modifications. This appears to happen during hepatocellular carcinoma (HCC) development in patients with liver cirrhosis, however, the accompanying transcriptional changes are virtually unknown. We investigated genome-wide transcriptional changes related to the senescence-to-immortality switch during hepatocellular carcinogenesis. Initially, we performed transcriptome analysis of senescent and immortal clones of Huh7 HCC cell line, and identified genes with significant differential expression to establish a senescence-related gene list. Through the analysis of senescence-related gene expression in different liver tissues we showed that cirrhosis and HCC display expression patterns compatible with senescent and immortal phenotypes, respectively; dysplasia being a transitional state. Gene set enrichment analysis revealed that cirrhosis/senescence-associated genes were preferentially expressed in non-tumor tissues, less malignant tumors, and differentiated or senescent cells. In contrast, HCC/immortality genes were up-regulated in tumor tissues, or more malignant tumors and progenitor cells. In HCC tumors and immortal cells genes involved in DNA repair, cell cycle, telomere extension and branched chain amino acid metabolism were up-regulated, whereas genes involved in cell signaling, as well as in drug, lipid, retinoid and glycolytic metabolism were down-regulated. Based on these distinctive gene expression features we developed a 15-gene hepatocellular immortality signature test that discriminated HCC from cirrhosis with high accuracy. Our findings demonstrate that senescence bypass plays a central role in hepatocellular carcinogenesis engendering systematic changes in the transcription of genes regulating DNA repair, proliferation, differentiation and metabolism.

## Introduction

Cellular senescence, a permanent loss of proliferative capacity despite continued viability and metabolic activity, is a critical feature of mammalian cells. It serves as a potent anti-tumor mechanism but it may also contribute to tissue aging [1], [2], [3]. This process was initially described in the form of replicative senescence [4], or telomere-dependent senescence, that has later been characterized as a DNA damage checkpoint response to the loss of telomere integrity, because of progressive shrinkage of the telomere DNA during cell replication [5]. Subsequently, telomere-independent or premature forms of senescence have been discovered. Thus, not only telomere attrition but also oncogene activation [6], tumor suppressor gene inactivation, as well as exposure to DNA-damaging agents can trigger senescence responses [1]. Cellular processes leading to a senescence-type of cell proliferation arrest are mediated mainly by p53 and p16^INK4A^-Rb signal transduction cascades [1], [7], [8]. Senescence response can be delayed or bypassed by experimental activation of telomerase reverse transcriptase (TERT), and/or inactivation of p53 and p16^INK4A^-Rb pathways. This leads cells to an immortal state with unlimited proliferation capacity [9]. Cells in pre-senescent, senescent and immortal states display highly divergent transcription patterns allowing them to exhibit distinct phenotypic and biochemical features [10], [11], [12].

Human tumors frequently exhibit TERT activation and inactivation of p53 and p16^INK4A^-Rb-mediated senescence control pathways leading on the postulation that gain of cellular immortality is one of their common features [13]. Hepatocellular carcinoma (HCC) cells are also believed to acquire immortality, particularly in patients with liver cirrhosis known to exhibit a senescent phenotype [14], [15]. Telomerase deficiency in mice accelerates the development of experimentally induced cirrhosis [16] and compromises liver regeneration [17]. The inactivation of c-*myc* or reactivation of p53 in murine HCC cells induces premature senescence leading to tumor regression [18], [19]. These findings infer that c-*myc* activation and p53 inactivation may serve as a means to overcome senescence control, at least in murine HCC tumors. Human liver cells do not express the TERT enzyme and exhibit moderate telomere shortening during aging, yet senescence markers usually remain negative in old liver tissues. During chronic hepatitis, the development of cirrhosis is associated with accelerated telomere shortening. Moreover, cirrhotic tissues exhibit strong senescence-associated β-galactosidase (SA-β-Gal) activity, suggesting that most hepatocytes in a cirrhotic liver display a senescent phenotype [20], [21], [22], [23]. In most human HCC tumors TERT expression is positive, telomerase activity is high and telomere length is short, but stabilized. However, a subset of HCC tumors display high SA-β-Gal activity suggestive of senescence arrest [14], [20], [24], [25], [26]. Thus, it appears that human HCC cells, as opposed to cirrhotic hepatocytes, acquire an immortal phenotype, although this has not yet been fully demonstrated. In favor of this suggestion, the genes encoding p53 and p16^INK4A^, two major players in senescence control, are known to be inactivated by mutation and/or epigenetic silencing in nearly 50% of HCCs [15]. However, several important questions remain unanswered with regard to the relevance of senescence escape or immortality in human HCC. Among others, (*i*) a comprehensive list of genes associated with hepatocellular senescence and immortality is lacking; (*ii*) the cellular processes associated with senescence-related changes in cirrhosis and HCC are not well-documented; (*iii*) the timing of senescence-to-immortality transition during HCC development is unknown; and (*iv*) the potential value of senescence-related gene signatures for the diagnosis and/or prognosis of HCC has not yet been assessed. A better understanding of these mechanisms could contribute significantly to the discovery of novel molecular targets for diagnosis and treatment of cirrhosis and HCC diseases, which account for more than 500,000 deaths each year [27].

Here, we applied an integrative functional genomics approach to explore the impact of senescence-related genes in liver cirrhosis and HCC. We first generated genome-wide expression profiles of *in vitro* hepatocellular senescence and immortality using a unique senescence model based on the reprogramming of replicative senescence in HCC-derived Huh7 cells [28]. By combined analysis of *in vitro*, *in vivo* and *in silico* data, we provide a comprehensive list of genes and cellular processes associated with hepatocellular senescence and gain of cellular immortality in humans. We also report on a robust 15-gene hepatocellular immortality signature test that can efficiently differentiate HCC from cirrhosis.

## Materials and Methods

### Huh7 Clones

The establishment and culture conditions of senescence-programmed C3 and G12, and immortal C1 and G11 clones have been described previously [28]. Briefly, HCC-derived Huh7 cells were transfected with pcDNA3.1 (Invitrogen) or pEGFP-N2 (Clontech) vectors to obtain C1 and C3, and G11 and G12 clones, respectively. Following transfection, single cell-derived colonies were selected by G-418 sulfate (500 µg/ml; Gibco) treatment under low-density clonogenic conditions. Senescence-programmed C3 and G12 clones proliferated stably until population doubling 80 (PD80) and PD90, respectively. Then, they entered senescence arrest as manifested by characteristic morphological changes, abundant SA-β-Gal staining and <5% 5-bromo-2′-deoxyuridine (BrdU) positivity after mitotic stimulation. Immortal C1 and G11 clones proliferated stably beyond PD140. For genome-wide expression studies described here, senescence-arrested C3 and G12 clones and immortal C1 and G11 clones were plated in triplicate onto 15-cm diameter petri dishes, left in culture for three days and collected for RNA extraction.

### Other Cells and Cell Lines

Freshly isolated human hepatocytes were obtained commercially (hNHEPS™- Human Hepatocytes, Lonza Group, Basel, Switzerland). Origin and culture conditions of HCC cell lines Huh7, HepG2, Hep3B, Hep40, PLC/PRF/5, SNU-387, SNU-398, SNU-423, SNU-449, SNU-475, FOCUS, Mahlavu and SK-Hep-1 were previously described [29]. The fibrolamellar HCC FLC4 cell line was provided by E. Galun (Hadassah, Israel) and cultivated as described for other HCC cell lines. MRC-5 human embryonic lung fibroblast cells (at PD45) were provided by R. Pedeux (Grenoble, France) and maintained in culture as previously described [30].

### Patients and Samples

Liver cirrhosis and HCC samples were collected from two medical centers in Turkey. Tissue samples were snap frozen in liquid nitrogen and stored at −80°C until use. Frozen tissues were cut into 20 µm thick slices, and scraped into microtubes for RNA extraction. Two 6 µm tissue slices were also cut for pathological examination.

### Ethics Statement

Clinical tissue sample collection was performed in accordance with a study protocol pre-approved by Ethical Committees of Ankara and Dokuz Eylul Universities, following written consent from each patient.

### RNA Extraction

Total RNA from cell lines and tissues was extracted using total RNA isolation kit (Promega, Madison, USA) and NucleoSpin RNA II Kit (MN Macherey-Nagel), respectively. DNase digestion was performed following kit instructions. Cell line and tissue RNA samples were analysed using Agilent Bioanalyzer.

### Genome-wide Gene Expression Profiling

Affymetrix platform with GeneChip Human Genome U133 Plus 2.0 arrays were used for microarray analysis of both cell and tissue RNA samples, following manufacturer instructions. GeneChip Operating Software (Affymetrix) was used to collect and store the microarray data. CEL files were uploaded to RMAExpress software to assess the quality of the arrays at the image level (http://rmaexpress.bmbolstad.com). Quality assessment of the Affymetrix datasets was performed using affyPLM (http://www.bioconductor.org). NUSE and RLE plots were drawn and outliers with high deviation from the average probe intensity value were excluded from further analyses. The microarray data reported in this paper have been deposited in the Gene Expression Omnibus (GEO) database under accession numbers of GSE17546 (Huh7 clones) and GSE17548 (cirrhosis and HCC tumor samples). All cell line clones, 15 cirrhosis and 15 HCC tumor samples passed RNA quality control (RNA Integrity Number, RIN>6.5) and microarray quality control tests. RMA normalization and class comparison analyses were performed using BRB-ArrayTools developed by Dr. Richard Simon and BRB-ArrayTools Development Team (http://linus.nci.nih.gov/BRB-ArrayTools.html; Version 4.2.0).

### Other Microarray Datasets

Two independent microarray datasets (GSE6764 and GSE19665) were downloaded from Gene Expression Omnibus (GEO) database (http://www.ncbi.nlm.nih.gov/geo) and analyzed by BRB Array tools after normalization with RMA.

### Gene Set Enrichment Analysis

Gene set enrichment analyses (GSEA) were performed using GSEA program of the Broad Institute [31]. For comparing *in vitro* and *in vivo* GSEA profiles based on “C2_ALL” curated gene list, a custom Matlab^©^ routine was applied to extract commonly enriched gene sets. Pearson's correlation coefficients were calculated using Matlab© and Fisher's exact test performed using VassarStats (Vassarstats.net).

### Cluster Analysis

Cluster 3.0 software [32] was used to assess unsupervised clustering of datasets. First, data were adjusted by centering genes and arrays separately based on mean values, and then the average linkage clustering was applied to genes and arrays using a correlation (uncentered) similarity metric. Cluster files were visualized by Java Treeview [33].

### Generation and Validation of a Senescence-based Genomic Classifier

A senescence-based genomic classifier associated with differential diagnosis of HCC from cirrhosis was generated in BRB Array Tools by Prediction Analysis of Microarrays [34] using data reported by Wurmbach et al. [35] as a “training set”. The resulting classifier was tested using the nearest template prediction (NTP) method [36] on a validation set constructed by combining data reported here for Turkish patients with data from Deng et al. [37] for Japanese patients. Nearest template prediction was performed using NTP module [36] of GenePattern program (http://www.broadinstitute.org/cancer/software/genepattern/) using default parameters of the module. The final image was generated using HeatMapImage module of the GenePattern and the output of the NTP.

### ATAD2 Expression Analysis by Quantitative PCR and Western Blot Analyses

ATAD2, one of the 15 genes identified as a senescence-based genomic classifier set was further analyzed for immortality-associated over-expression in 14 HCC cell lines, as compared to freshly isolated human hepatocytes and MRC-5 human embryonic lung fibroblast cells (PD44) at RNA and protein levels. ATAD2 RNA expression was compared by quantitative real-time PCR as previously described [38], using a specific primer pair (forward: 5′-AGG CTC ATT GGA AAA ACC T-3′; reverse: 5′-CCT GCG GAA GAT AAT CGG TA-3′). GAPDH was tested as a housekeeping control gene using the following primers: forward: 5′-GGC TGA GAA CGG GAA GCT TGT CAT-3′; reverse: 5′-CAG CCT TCT CCA TGG TGG TGA AGA-3′. The relative expression of ATAD2 RNA in cell lines was calculated as compared to that of normal hepatocytes. ATAD2 protein expression was compared by western blot analysis of cell lysates, as described previously [38], except that an anti-ATAD2 rabbit polyclonal antibody (Sigma; cat. no: HPA019860) was used at 1∶500 dilution as the primary antibody. Anti-calnexin rabbit polyclonal antibody (Sigma; cat. no: C4731) was used at 1∶10000 dilution for the loading control. The specificity of anti-ATAD2 antibody was validated by western blot analysis in Hep3B cells after transfection with ATAD2-siRNA1 described by Caron et al. [39]. For comparative analysis of ATAD2 protein expression between immortal and senescent cells, senescence was induced in Huh7 cells by Adriamycin (0.1 µM) treatment for three days as previously described [40]. Briefly, Adriamycin- and DMSO vehicle control-treated cells were maintained in culture for three days. After confirming the senescence induction by morphological examination and SA-β-Gal staining, cell lysates were subjected to western blot analysis.

## Results

### Study Design

**Figure 1:**
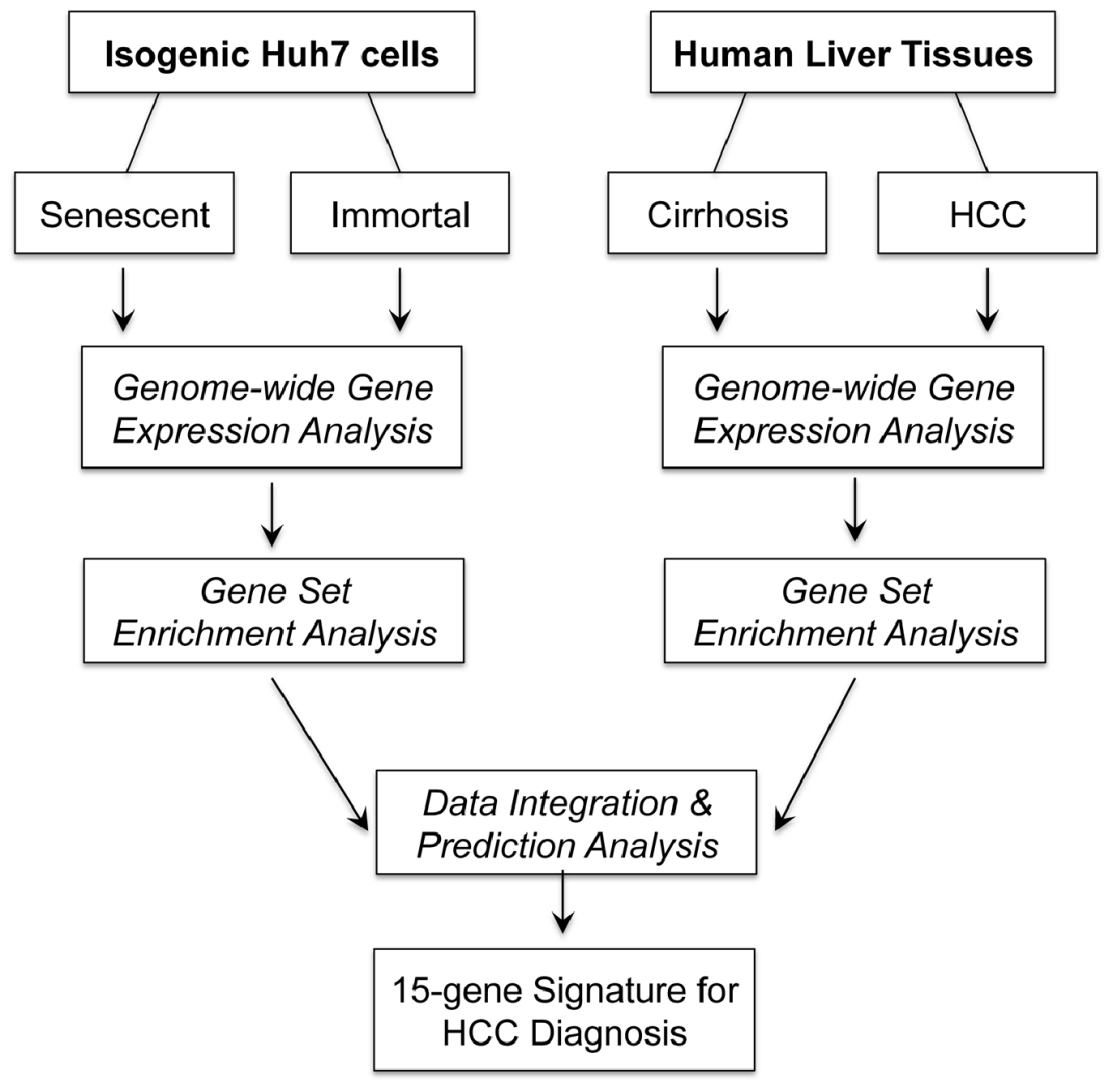
Flow chart summarizing the study design. We analyzed genome-wide gene expression in isogenic Huh7 clones with senescent and immortal phenotypes, as well as in cirrhotic tissues and HCC tumors. Both sets of data were subjected to GSEA using “C2-All” curated gene sets (“C2_All”) of molecular signature database (MSigDB; www.broadinstitute.org/gsea/). In order to assess senescence- and immortality-related gene expression changes during hepatocellular carcinogenesis, genes differentially expressed in the model of cellular senescence and immortality were identified and the evolution of their expression profiles in pre-neoplastic and neoplastic liver lesions were examined. Finally, a 15-gene senescence-based signature was generated using a training set of cirrhosis and HCC samples, and validated using independently generated test datasets.

In order to analyze the participation of senescence-related genes in human liver diseases, we designed a study protocol, as outlined in Fig. 1. First, we generated genome-wide expression profiles of Huh7 cell-line derived isogenic clones, as well as cirrhosis and HCC tissues. The isogenic Huh7 clones that we used differed from each other by their entry into replicative senescence arrest (at PD80 to PD90) or lack of it (beyond PD150), resulting in a major shift in tumorigenicity [28]. Next, we subjected *in vitro* and *in vivo* gene expression data to gene set enrichment analysis (GSEA) to identify and compare functional groups of genes associated with senescence *versus* immortality, and cirrhosis *versus* HCC. Furthermore, we integrated our *in vitro* data with publicly available *in vivo* data for a senescence-based comparison of progressive liver lesions associated with hepatitis C virus (HCV)-induced HCC, and established a senescence-based gene signature test for differential diagnosis of HCC.

### Gene Expression Profiles of Hepatocellular Senescence in vitro

**Figure 2:**
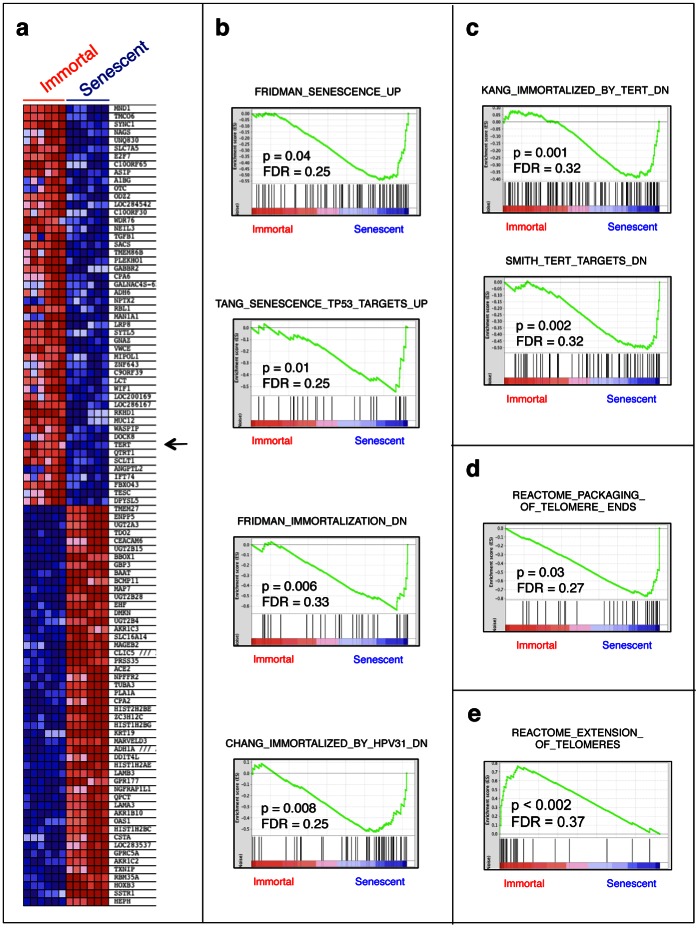
Gene expression profile analysis by gene set enrichment analysis assay (GSEA) established that senescent and immortal Huh7 clones displayed differential expression of previously identified senescence- and immortality-associated gene sets respectively, as well as those regulating telomere maintenance. (**a**) Heat map representation of the top 100 deregulated genes in immortal Huh7 clones (Immortal) *versus* senescent Huh7 (Senescent) clones. Red: up-regulated; blue: down-regulated; arrow indicates TERT gene whose expression is down-regulated in senescent clones. Previously identified gene sets (available at molecular signature database (MSigDB; www.broadinstitute.org/gsea/) were screened to identify those that are differentially enriched in senescent or immortal Huh7 clones by the analysis of their relative expression levels using GSEA method. (**b**) Gene set enrichment plots showing the up-regulated expression of two previously known senescence-associated gene sets in senescent Huh7 clones, including genes that are commonly up-regulated in senescent cells (“FRIDMAN_SENESCENCE_UP”) [10] and p53-responsive genes up-regulated during replicative senescence arrest (“TANG_SENESCENCE_TP53_TARGERTS_UP”) [41]. In addition, genes known to be down-regulated during immortalization in general (“FRIDMAN_IMMORTALIZATION_DN”) [10], and by human papillomavirus 31 (“CHANG_IMMORTALIZED_BY_HPV_DN”) [42] were also up-regulated in senescent Huh7 clones. (**c**) Genes known to be down-regulated by TERT-mediated immortalization (“KANG_IMMORTALIZED_BY_TERT_DN”) [43] and TERT-repressed target genes (“SMITH_TERT_TARGETS_DN”) [44] were also enriched in senescent Huh7 clones. (**d**) Genes involved in telomere end packaging (“REACTOME_PACKAGING_OF_TELOMERE_ENDS”; www.reactome.org) were upregulated in senescent Huh7 clones. (**e**) In contrast, genes involved in telomere extension (“REACTOME_EXTENSION_OF_TELOMERES”; www.reactome.org) were enriched in immortal Huh7 clones. Enrichment scores (ES) are shown on the y-axis. Positive and negative ES indicate enrichment in immortal and senescent Huh7 clones, respectively. X-axis bars represent individual genes of the indicated gene sets. FDR: False discovery rate, p: nominal p-value. Three biological replicates from each clone were analyzed for genome-wide gene expression using Affymetrix 54 K microarrays and normalized data were used for gene set enrichment analysis (GSEA).

We profiled four independently established Huh7 clones, subdivided into senescent and immortal phenotypes (i.e., two clones from each phenotype) using gene expression analysis with pangenomic 54 K Affymetrix microarrays. Three independent biological replicates from each clone were used so that a total of 12 gene chips were performed. Hierarchical clustering of expression values of the top 50 down- and up-regulated genes of each phenotype segregated cell samples based on phenotypic assignment, which suggested a common transcriptional consequence of a switch between senescent and immortal fates (Fig. 2a). Next we calculated GSEA enrichment scores [31] for six senescent cell samples against six immortal cell samples using all curated gene sets (“C2_All”) available at molecular signature database (MSigDB; www.broadinstitute.org/gsea/). Based on significant nominal P values (P<0.05), senescent and immortal phenotypes were enriched in 598 and 113 gene sets, respectively (Data S1).

Enriched gene sets included those participating in senescence- and immortality-related cellular processes and pathways, providing evidence for transcriptional validation of senescent and immortal phenotypes of Huh7 clones (Figs. 2b–e). As shown in Fig. 2b, senescent Huh7 cells were enriched in gene sets that are commonly up-regulated in senescent cells (“FRIDMAN_SENESCENCE_UP”) [10]. In addition, p53-responsive genes up-regulated during replicative senescence arrest (“TANG_SENESCENCE_TP53_TARGERTS_UP”) [41], as well genes down-regulated during immortalization in general (“FRIDMAN_IMMORTALIZATION_DN”) [10], and by human papillomavirus 31 (“CHANG_IMMORTALIZED_BY_HPV_DN”) [42], were also up-regulated in senescent Huh7 clones. Interestingly, four enriched gene sets were connected directly with either TERT or telomeres (Fig. 2c–e). Genes down-regulated by TERT-mediated immortalization (“KANG_IMMORTALIZED_BY_TERT_DN”) [43], as well as TERT-repressed target genes (“SMITH_TERT_TARGETS_DN”) [44], were enriched in senescent Huh7 clones (Fig. 2c). Furthermore, genes involved in telomere end packaging (“REACTOME_PACKAGING_OF_TELOMERE_ENDS”; www.reactome.org) were up-regulated in senescent Huh7 clones (Fig. 2d), while genes involved in telomere extension (“REACTOME_EXTENSION_OF_TELOMERES”; www.reactome.org) were enriched in immortal Huh7 clones (Fig. 2e).

### Association of Cirrhosis and Hepatocellular Carcinoma with Senescent and Immortal Phenotypes Respectively

**Figure 3:**
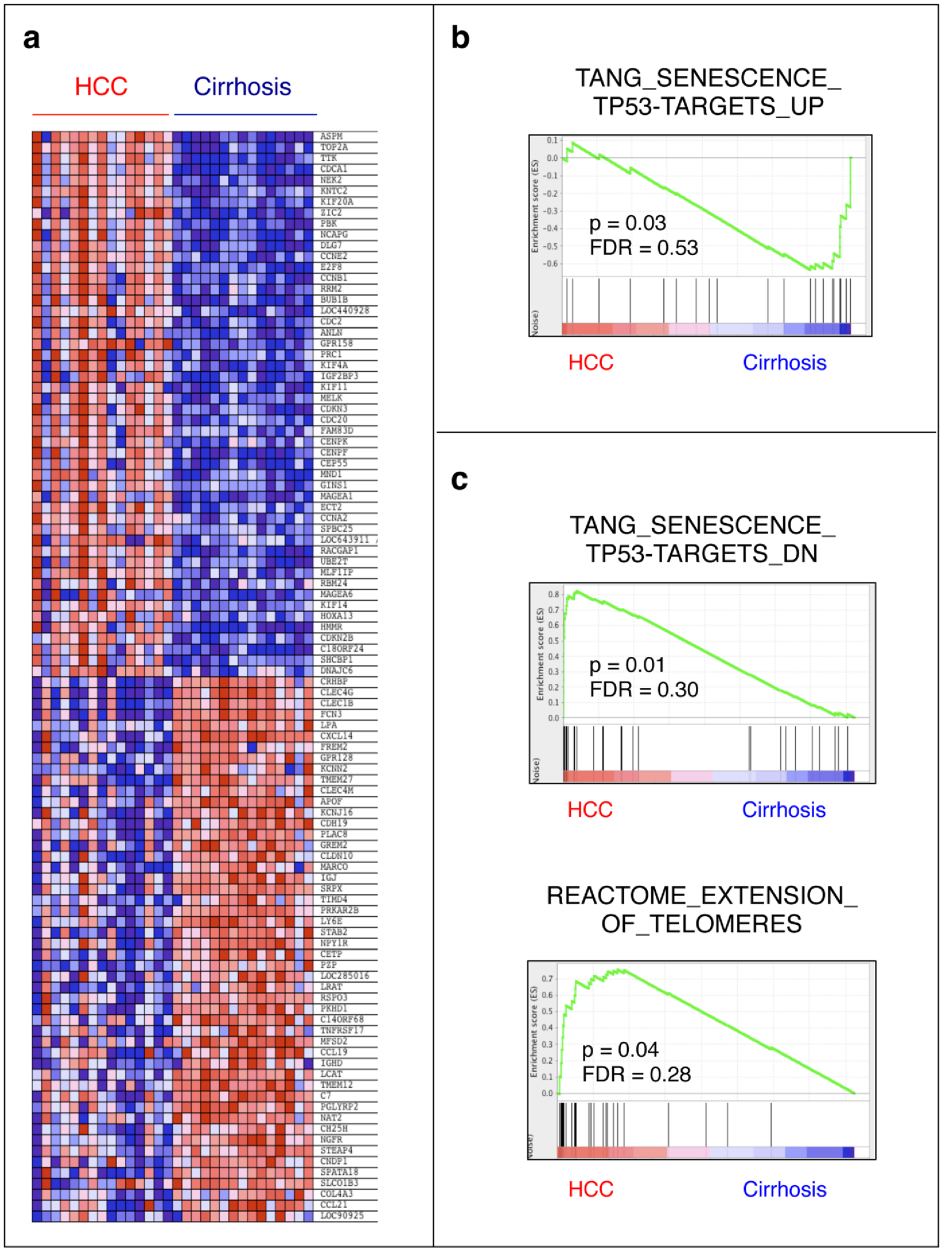
Gene expression profile analysis by gene set enrichment analysis (GSEA) revealed the overexpression of senescence-upregulated genes in cirrhosis, but over-expression of senescence-downregulated genes and telomere extension genes in HCC tissues. (**a**) Heat map representation of the top 100 deregulated genes in hepatocellular carcinoma (HCC) *versus* cirrhosis samples. Red: up-regulated; blue: down-regulated. Previously identified gene sets (available at molecular signature database (MSigDB; www.broadinstitute.org/gsea/) were screened to identify those that are up-regulated in cirrhosis or HCC tissues by the analysis of their relative expression levels using GSEA method. (**b**) Enrichment plot of p53-responsive genes up-regulated during replicative senescence arrest (“TANG_SENESCENCE_TP53_TARGERTS_UP”) [41] showing over-expression in cirrhosis. (**b**) In contrast, p53-responsive genes down-regulated during replicative senescence arrest (“TANG_SENESCENCE_TP53_TARGERTS_DN”) [41] and those involved in telomere extension (“REACTOME_EXTENSION_OF_TELOMERES”; www.reactome.org) were overexpressed in HCC tumors. Enrichment scores (ES) are shown on the y-axis. Positive and negative ES indicate enrichment in HCC and cirrhosis samples, respectively. X-axis bars represent individual genes of the indicated gene sets. FDR: False discovery rate, p: nominal p-value. Fifteen cirrhosis and fifteen HCC samples were analyzed for genome-wide gene expression using Affymetrix 54 K microarrays and normalized data were used for gene set enrichment analysis (GSEA).

According to the protocol described in Fig. 1, next we performed global gene expression analysis of 30 liver tissues, including 15 cirrhosis and 15 HCC samples. All HCC samples used in this study were obtained from cirrhotic patients (Table S1). Hierarchical clustering with 50 most up- and down-regulated genes were identified by GSEA-segregated tissue samples according to their clinical phenotypes (Fig. 3a). Gene set enrichment analysis of this *in vivo* data set using “C2_All” gene sets determined that cirrhosis and HCC phenotypes are associated with 161 and 189 enriched gene sets, respectively (nominal P values<0.05; Data S1). Among those gene sets, several were connected to senescence- and immortality-related events. For example, cirrhosis was enriched in p53-responsive genes up-regulated during replicative senescence arrest (“TANG_SENESCENCE_TP53_TARGETS_UP”) [41] (Fig. 3b). In contrast, HCC was enriched in p53-responsive genes down-regulated during replicative senescence arrest (“TANG_SENESCENCE_TP53_TARGETS_DN”) [41] (Fig. 3c-top). Hepatocellular carcinoma tumors were also enriched in the expression of genes involved in telomere extension (“REACTOME_EXTENSION_OF_TELOMERES”; www.reactome.org) (Fig. 3c-down).

### Senescence-related Gene Networks in Cirrhosis and Hepatocellular Carcinoma

**Figure 4:**
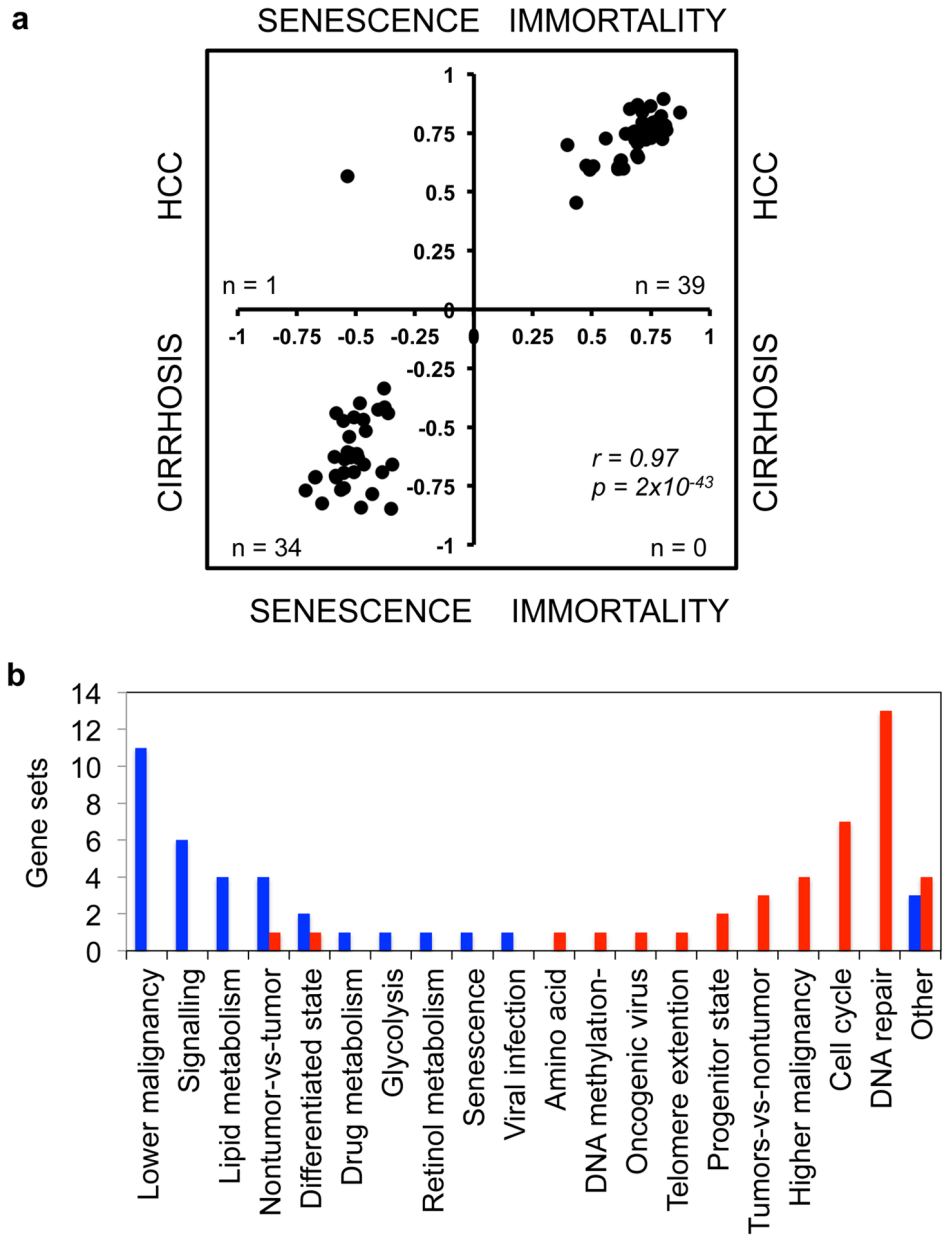
Comparative analysis of gene sets enriched in Huh7 clones and diseased liver tissues associated cirrhosis with senescence and HCC with immortality phenotypes, respectively. (**a**). This analysis revealed also that cirrhosis/senescence- and HCC/immortality-associated gene sets implicated distinct biological features specific to each phenotype (**b**). (**a**) Scatter plot compares enrichment scores of 74 gene sets commonly enriched in Huh7 clones (senescent or immortal) and diseased liver tissues (cirrhosis or HCC) with a P value less than 0.05. Thirty-nine gene sets (53%) were significantly enriched in both HCC and immortal samples whereas 34 (46%) gene sets were significantly enriched in both cirrhosis and senescent samples (correlation value r = 0.97, p = 2×10^−43^). Only one gene set (1%) was enriched in both HCC and senescent clones. (**b**) Distribution of biological features defined by different gene sets in cirrhosis/senescence (blue columns) and HCC/immortality (red columns) phenotypes, respectively.

The comparison of cell line and tissue enrichment scores based on commonly enriched gene lists (Data S1) revealed a striking correlation between senescence and cirrhosis (P<10^−115^; r = 0.35), as well as, between immortality and HCC (P = 8×10^−114^; r = 0.72). This finding suggested that many functional gene clusters overexpressed in cirrhosis and HCC were directly related to the senescent and immortal phenotypes, respectively. To further investigate this interesting correlation, we selected gene sets that are co-enriched in four groups of biological samples (i.e. senescent cells, immortal cells, cirrhotic tissues and HCCs) with a nominal P-value less than 0.05. As shown in Fig. 4a, 34 of 74 common gene sets (46%) were co-enriched in senescent cells and cirrhotic tissues, whereas 39 (53%) were co-enriched in immortal cells and HCCs (Two-tailed Fisher exact test, P = 2.6×10^−20^). Pearson correlation values of co-enrichment scores were also significant (r = 0.97, P = 2×10^−43^; Data S1). Gene sets up-regulated in cirrhosis/senescence group were also up-regulated in non-tumor tissues as opposed to those with tumors (four gene sets), or in less malignant tumors *versus* more malignant tumors (11 gene sets). In contrast, genes up-regulated in HCC/immortality group were associated with tumors as opposed to non-tumor tissues (four out of five gene sets), or in more malignant tumors as compared to less malignant tumors (four gene sets). The HCC/immortality state was characterized by an up-regulation of genes involved in DNA repair (13 gene sets), cell cycle (seven gene sets), progenitor state (two gene sets), telomere extension, DNA methylation and branched chain amino acid metabolism. In contrast, genes involved in cell signaling (six gene sets), lipid metabolism (four gene sets), drug metabolism, retinol metabolism and glycolysis were down-regulated (Fig. 4b).

**Figure 5:**
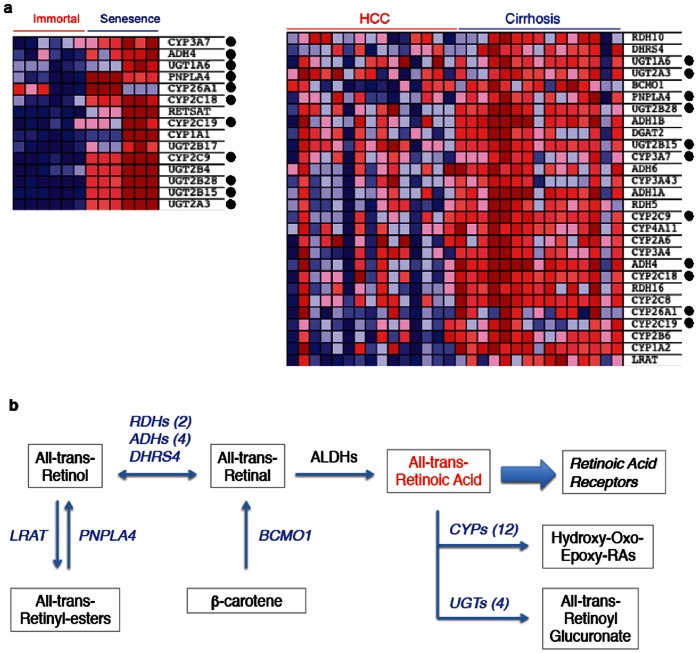
Comparative analysis of core enriched gene sets in Huh7 clones (senescent *versus* immortal) and diseased liver tissues (cirrhosis *versus* HCC) indicated that retinoid metabolism genes (“KEGG_RETINOL_METABOLISM”) undergo systematic changes in immortal cells and HCC, when compared to senescent cells and cirrhosis, respectively. (**a**) Heat map of core enriched retinoid metabolism genes in Huh7 clones (left) and diseased liver tissues (right). Red: up-regulated; blue: down-regulated. Genes commonly deregulated in both Huh7 clones and diseased liver tissues are indicated with a dot. (**b**) A simplified view of retinoid metabolism. Enzyme-encoding genes down-regulated in HCC are shown in blue. LRAT: lechitin retinol acetyl transferase, PNPLA4: patatin-like phospholipase domain containing-4, RDHs: retinol dehydrogenases; ADHs: alcohol dehydrogenases; DHRS4: dehydrogenase/reductase (SDR family) member-4; BCMO1: beta-carotene 15,15′-monooxygenase-1; CYPs: Cytochrome P-450 family proteins; UGTs: UDP glucoronosyltransferases.

Detailed analysis of genes involved in retinoid metabolism [45], [46] revealed that the expression of several genes encoding critical enzymes catalyzing the synthesis of retinoic acid (the active form of retinoids) was down-regulated in HCC tumors as compared to cirrhotic liver tissue. There was also down-regulated expression of genes involved in the storage of retinoids in tumors. Down-regulated genes included two members of retinol dehydrogenases, four members of alcohol dehydrogenases, NADP(H)-dependent retinol dehydrogenase/reductase (*DHRS4*) and β-carotene 15,15′-monooxygenase 1 (*BCMO1*), which are all involved in the synthesis of retinal, the immediate precursor of retinoic acid. Two genes involved in the synthesis of storage retinyl esters, namely lecithin:retinol acyltransferase (*LRAT*) and patatin-like phospholipase-4 (*PNPLA4*) were also down-regulated in HCC cells (Fig. 5).

### A Senescence-to-immortality Switch between Dysplasia and Hepatocellular Carcinoma

**Figure 6:**
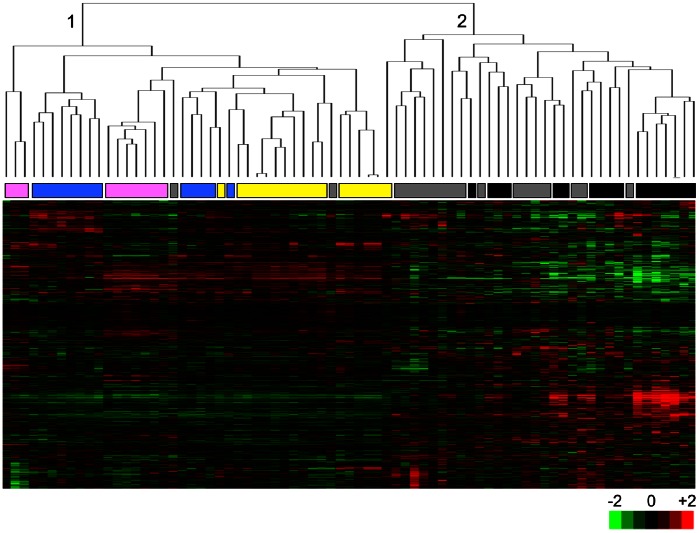
Hierarchical clustering of 75 non-malignant and malignant liver tissue samples using 1813 senescence-associated gene probe sets. Hepatocellular carcinoma and non-tumor liver tissues formed two distinct clusters (1 and 2) with the exception of one dysplasia and two early HCC samples. The rows and columns represent genes and samples, respectively on the cluster map. Tissue samples are normal liver (pink), cirrhosis (blue), dysplasia (yellow), early HCC (gray), and advanced HCC (black). Red: over-expressed, green: under-expressed probe set in the heat map.

Hepatocellular carcinogenesis is a multi-step process that is usually manifested by progressive histological changes in the liver from the cirrhosis stage to dysplasia followed by HCC [47]. Based on close association of cirrhosis with senescence and that of HCC with immortality, we hypothesized that the relative expression of senescence- and immortality-associated genes in different liver lesions may serve as a powerful means to dissect the timing of transition from a senescent state to an immortal phenotype during hepatocellular carcinogenesis. With this aim, we first generated a list of “senescence-related genes” by comparing differential gene expression between senescent and immortal Huh7 clones. Then, we analyzed the expression patterns of these “senescence-related genes” in a spectrum of hepatic lesions representing different steps of HCC development. The list of senescence-related genes was established by class comparison analysis of *in vitro* gene expression data. Multivariate permutation tests identified 1220 genes represented by 1813 probe sets with statistically significant expression changes between senescent and immortal clones (*P-values*<10^−7^; fold-changes between senescent and immortal clones: >2.0). The selected probe sets were then tested against a publicly available gene expression dataset for tissues at different histological stages of HCC development in HCV patients [35]. The tissue set was composed of 10 normal liver samples, 13 cirrhotic tissues, 17 dysplastic lesions (originally described low- and high-grade dysplasia cases combined), 17 early HCCs (originally described very early and early HCC cases combined) and 18 advanced HCCs (originally described advanced and very advanced cases combined). Unsupervised clustering analysis applied to compare these hepatic tissue samples (n = 75 in total) generated two major clusters. Cluster 1 grouped together 39 out of the 40 non-HCC samples (97.5%) and 1 out of the 35 (3%) HCC samples. Conversely, cluster 2 was composed of 34 out of the 35 HCCs (97%) and one of 40 (2.5%) of the non-HCC samples (Fig. 6). Dysplastic lesions together with a subset of cirrhosis tissues formed a homogenous subgroup under cluster 1, while normal liver samples shared similarities with either cirrhotic or dysplastic tissue. HCC samples formed several minor clusters, with a tendency of early and advanced tumors to form distinct sub-clusters. The most interesting finding of this analysis was the clustering of dysplastic liver lesions together with cirrhosis samples that are in a senescent-like state rather than with HCC samples assigned to an immortal-like state.

### Fifteen-gene Hepatocellular Immortality Signature for Diagnosis of Hepatocellular Carcinoma

**Figure 7:**
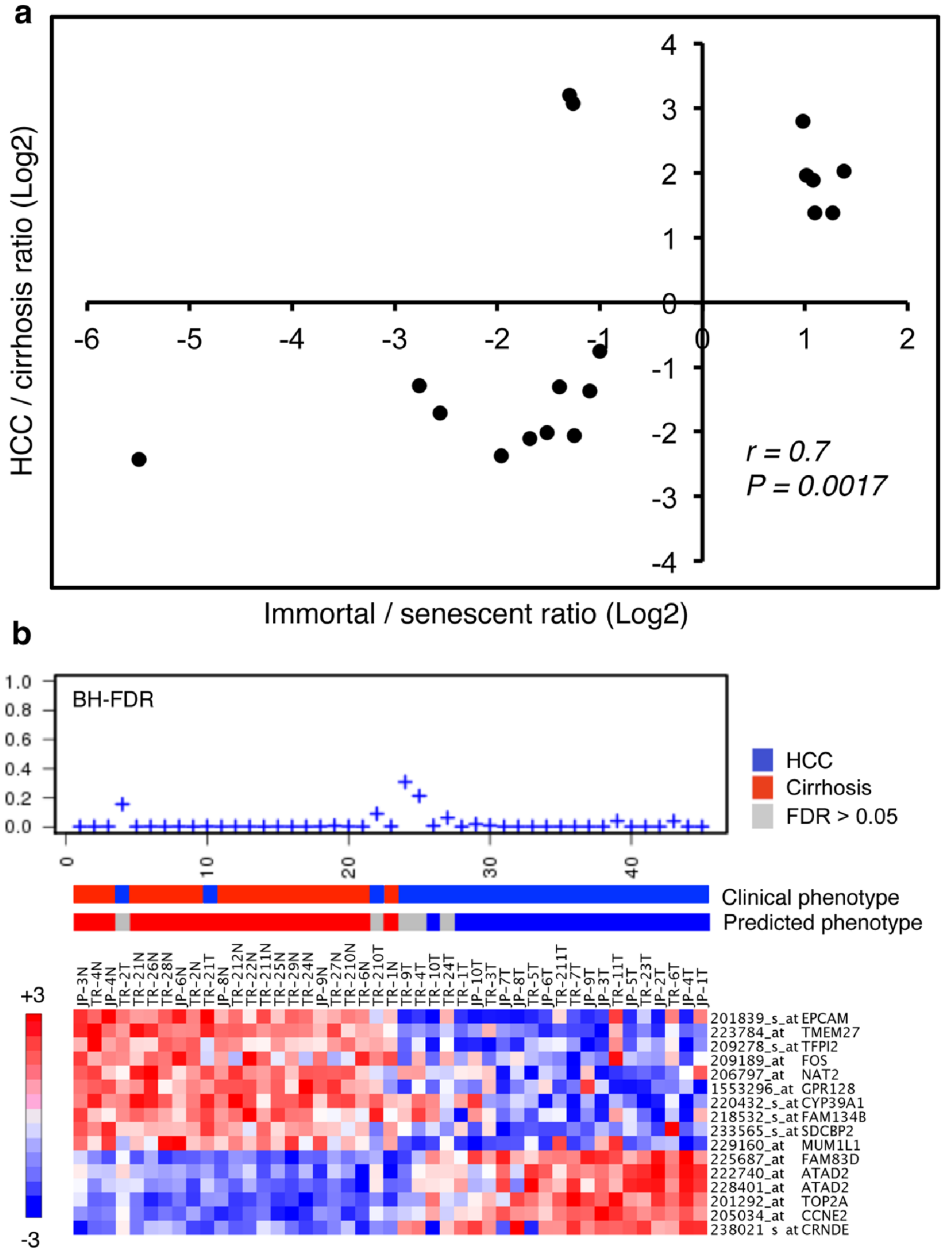
Generation and validation of a senescence-based gene classifier for differential diagnosis of cirrhosis and HCC. (**a**) Scatter plot graphic compares relative expression levels (Log2 ratios) of 18 classifier probe sets representing 17 genes in Huh7 clones (immortal *versus* senescent) and diseased liver tissues (HCC *versus* cirrhosis). Expression ratios of classifier genes showed a linear correlation (correlation value r = 0.7, p = 0.0017) with ratios observed in Huh7 clones (immortal/senescent) and diseased liver tissues (HCC/cirrhosis). The classifier set was identified by PAM analysis of 1813 senescence-associated probe sets using a training tissue set composed of cirrhosis (n = 13) and HCC (n = 35) samples described by Wurmbach et al. [35]. Two probe sets which did not show expression patterns compatible with our *in vivo* senescence model were discarded to define a final signature set composed of 16 probe sets representing 15 genes. (**b**) Validation of molecular prediction of HCC and cirrhosis by 15-classifier gene set. Using the nearest template prediction method [36], we compared expression levels of sixteen probe sets representing 15 classifier genes in a test tissue set composed of 20 cirrhosis and 25 HCC samples originating from Turkish (TR) patients described in this report, and Japanese (JP) patients described elsewhere [37]. BH FDR (Benjamini-Hochberg false discovery rates) values (top), clinical *versus* predicted phenotypes (middle) and heatmaps of classifier gene expression levels (bottom) are shown. The test provided a diagnostic result for 40 out of 45 samples (89%) with 97.5% (39/40) accuracy.

Based on remarkable clustering of tumor and non-tumor tissues by the 1220 senescence-related genes, we then asked whether we could select a smaller subset for discrimination of HCC from cirrhosis. We used expression data from 35 HCC and 13 cirrhosis samples from Wurmbach et al. [35] as a “training set”. A PAM analysis using “nearest shrunken centroid method” [34] identified 18 classifiers, composed of six immortality-associated probe sets (representing five genes) up-regulated in HCC tissues, ten senescence-associated probe sets up-regulated in cirrhosis samples, and two senescence-associated probe sets up-regulated in HCC (Fig. 7a). Fisher's exact test demonstrated a strong association of cirrhosis with senescence and HCC with immortal phenotypes (P = 0.0015). Then, we selected ten “cirrhosis- and senescence- associated” and five “HCC- and immortality-associated” genes (16 probe sets in total) to construct a “hepatocellular immortality signature set” (Table S2). Next, we tested the diagnostic value of the signature genes using a “test set” composed of 45 tissue samples, including 30 Turkish patient samples reported here and 15 Japanese patient samples with publicly available expression data [37]. Based on Nearest Template Prediction method [36], the signature set was able to predict 100% (20/20) of cirrhotic tissues with high confidence (FDR<0.05). Five of 25 HCC samples (20%) were unpredictable (FDR>0.05). Of the remaining 20 HCC samples, 19 (95%) were predicted correctly (Fig. 7b). Overall, the signature set provided high confidence prediction (FDR<0.05) in 89% (40/45) of patients with 97.5% (39/40) accuracy.

### Association of ATAD2 RNA and Protein Expressions with HCC and Cellular Immortality

**Figure 8:**
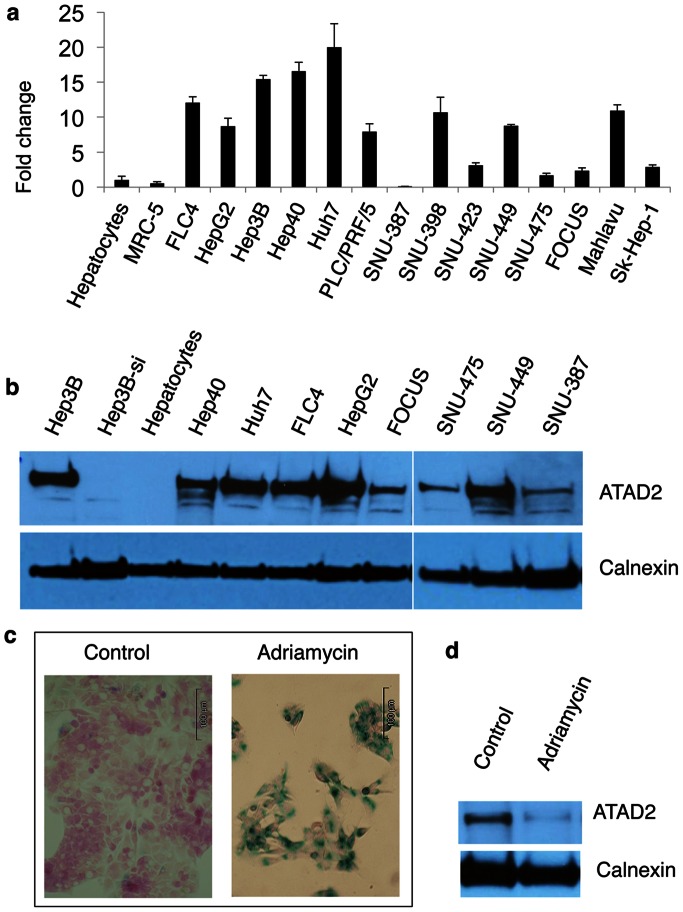
Association of ATAD2 RNA and protein expressions with HCC and cellular immortality. (**a**) Amplified expression of ATAD2 RNA in HCC cell lines, as compared to normal hepatocytes and MRC-5 fibroblasts. Total RNAs were extracted from freshly isolated adult human hepatocytes (Hepatocytes), MRC-5 human embryonic lung fibroblast cells (PD44) and 14 HCC cell lines; reverse transcribed into cDNA; and ATAD2 RNA was quantified by quantitative real-time PCR using specific primers. ATAD2 expression values for each sample were normalized with housekeeping gene GAPDH RNA values. Relative expression of ATAD2 in MRC-5 and HCC cell lines was expressed in reference to its expression in hepatocytes. Averages of three measurements. Error bars: SD. (**b**) Amplified expression of ATAD2 protein in HCC cells, as compared to normal hepatocytes. Total proteins were extracted from freshly isolated adult human hepatocytes (Hepatocytes), untreated (Hep3B) and ATAD2 siRNA1-treated (Hep3B-si) Hep3B and eight other HCC cell lines, and ATAD2 protein levels were tested by western blot analysis using a specific anti-ATAD2 antibody (ATAD2). Western blot analysis of calnexin protein from the same blots was used for loading control (Calnexin). (**c, d**) Comparative analysis by western blotting demonstrated that ATAD2 protein is overexpressed in immortal Huh7 cells as compared to senescence-arrested Huh7 cells. (**c**) Huh7 cells were treated with Adriamycin (0.1 µM) or DMSO (Control) for three days and subjected to senescence assay by SA-β-Gal staining (blue). Cells were counterstained with fast red (red). (**d**) Total protein was extracted from control and Adriamycin-treated Huh7 cells, and ATAD2 and Calnexin proteins were tested as described in (**b**).

The *ATAD2*, one of the fifteen hepatocellular immortality signature genes, was of particular interest warranting further investigation. The ATAD2 gene is mapped to chromosome 8q24 and codes for a predicted protein of 1,391 amino acids that contains a double AAA ATPase domain and a bromodomain [48]. The 8q24 locus displays frequent copy number gains in HCC [49], and many other cancers [50]. Therefore, we selected ATAD2 as a representative of our hepatocellular immortality signature to validate its immortality- and HCC-associated expression by additional experiments (Fig. 8). Freshly isolated normal adult human hepatocytes and MRC-5 normal human fetal lung fibroblasts (at PD44) were used as non-immortal control cells that enter replicative senescence at around PD65 [30]. When compared to normal hepatocytes, most HCC cell lines (n = 12/14; 86%) displayed between two- and 20-fold higher ATAD2 mRNA expression. ATAD2 expression was less in MRC-5 cells than hepatocytes (Fig. 8a). In order to further investigate ATAD2 expression, we tested its protein levels using a polyclonal rabbit anti-ATAD2 antibody that recognized a single major band in Hep3B HCC cells (Fig. 8b line Hep3B). The knock-down of ATAD2 by siRNA1 [39] in these cells resulted in the loss of an anti-ATAD2 immunoreactive band (Fig. 8b line Hep3B-si), demonstrating the specificity of this antibody. ATAD2 protein was undetectable in normal hepatocytes, but highly abundant in six out of nine HCC cell lines, and easily detectable in the remaining three (Fig. 8b). In order to further investigate immortality-associated expression of ATAD2 in HCC cells, we induced senescence arrest in Huh7 cells by 0.1 µM Adriamycin treatment (Fig. 8c) as previously described [40], and compared ATAD2 expression between Adriamycin-treated and control Huh7 cells by western blot assay. We observed a drop in the levels of ATAD2 proteins in senescence-arrested cells, as compared to immortal Huh7 cells (Fig. 8d).

## Discussion

Cellular senescence, considered for a long time to be an *in vitro* phenomenon, emerged in recent years as a critical mechanism that may play key roles in tissue aging as well as in the development of different tumor types [1]. Here, we used a unique *in vitro* hepatocellular senescence model to map senescence-related events associated with *in vivo* HCC development. Our *in vitro* model displayed a gene expression pattern compatible with replicative senescence and TERT-induced cellular immortalization, in conformation of our previously published observations [28]. We were fortunate to find a high number of differentially expressed genes between senescent and immortal clones that served as an investigational tool to examine senescence-related transcriptional events occurring during hepatocellular carcinogenesis. Based on this, we provide here transcription-based evidence that cirrhosis and HCC represent two opposite cellular phenotypes, senescence and immortality, respectively. One of the major features of this phenotypic opposition was the status of telomere maintenance genes both between senescence and immortality, and cirrhosis and HCC (Figs. 2, 3). The activation of TERT and telomere end extension genes in immortal and HCC phenotypes is of particular interest. Accelerated shortening of telomeres associated with a lack of telomerase activity and high cell turnover during chronic hepatitis has been recognized as a hallmark of cirrhosis several years ago [16], [21], [51]. More recently, constitutional “loss-of-function” type of telomerase (*TERT* or *TERC* genes) mutations have been identified as a risk factor for cirrhosis [52], [53]. In contrast to cirrhosis, HCC is known to reactivate TERT expression [54], display high telomerase activity [24] and stabilize telomeres [24], [55]. Based on our present data supported by these earlier reports, we propose that the activation of telomerase activity is a key event for the gain of immortalized phenotype by HCC cells. While working on this manuscript, recurrent and activating *TERT* promoter mutations have been reported for HCC cell lines [56], in strong support of our hypothesis.

The transition from a senescent state to an immortal state coincided with early HCC lesions while dysplastic lesions remained associated with cirrhosis and normal liver sample groups indicating a non-immortal state. This pattern correlates with malignant transformation in other tissues where pre-neoplastic lesions display a senescent state from which neoplastic transformation emerges with a gain of phenotypic and molecular features that are linked to an immortal state [57].

Co-enrichment of a high number of gene sets in cirrhotic tissues and senescent cells as well as in HCCs and immortal cells was highly interesting. This finding further emphasized the biological evidence for a gain of immortal phenotype in human HCC. Among the gene sets co-enriched in HCC and immortal cells, cell cycle and DNA repair gene sets were at the top of the list (Fig. 4b). Up-regulation of cell cycle and DNA repair genes in HCC is already known [35], [58]; and the overexpression of cell cycle genes in immortal cells is expected. The up-regulation of DNA repair genes may serve as a mechanism to escape from DNA damage-induced senescence arrest by increasing DNA repair capacity of immortal or HCC cells.

Another interesting outcome of co-enrichment analysis was the differential association of metabolism regulatory gene sets with cirrhosis/senescence and HCC/immortality phenotypes. Co-enrichment patterns revealed that genes involved in glycolysis as well as those regulating drug, lipid and retinol metabolisms were down-regulated in both immortal cells and HCC tumors. Down-regulation of genes encoding the enzymes necessary for retinoic acid biosynthesis and intracellular retinoid storage in HCC is of particular interest. Retinoic acid, which is the active metabolite of retinoids, regulates a wide range of biological processes including development, differentiation, proliferation, and apoptosis [59]. Normal hepatocytes together with hepatic stellate cells play an indispensable role in the availability of retionic acid and the storage of dietary retinoids [46]. Deregulated expression of retinoid metabolism genes in HCC is expected to cause a deficit in the synthesis of retinoic acid as well as in the storage of its metabolic precursors (Fig. 5). Accordingly, reduced retinoid content has been reported for HCC [46], [60], [61]. A deficit in cellular retinoic acid levels in HCC cells, due to the expression changes reported here, may cause severe perturbations in a multitude of cellular processes governed by retinoic acid [59] by conferring a survival advantage to immortalized HCC cells. Thus the restoration of retinoic acid availability in HCC cells may adversely affect their survival. In favor of this hypothesis, treatment with a synthetic analog of retinoic acid successfully prevented second primary tumors in post-surgical HCC patients [62]. Thus, a deficit in the availability of endogenous retinoic acid might facilitate malignant transformation and tumor progression.

The most important risk factor for HCC is cirrhosis that is present in 80 to 90% of patients with HCC. The patients with cirrhosis develop HCC with a rate of 1.4–3.3% per year [27]. Therefore, the screening of cirrhotic patients by ultrasonography of the liver combined with measurement of serum alpha-fetoprotein levels every 6 to 12 months for HCC development is recommended. However, the strength of the evidence supporting the efficacy of surveillance is modest [63]. To overcome the lack of efficacy, molecular HCC diagnosis techniques have been proposed [64]. Previously reported molecular techniques used either candidate genes [65] or genome-wide expression data [35], [54], [66], [67] to discriminate HCC from cirrhosis or dysplasia. None of these molecular tests have yet to enter into surveillance recommendations [64], probably because their prediction strength did not reach the required level and/or they require simultaneous analysis of dozens, even hundreds, of genes. Here, we provide a highly promising hepatocellular immortality signature test for HCC diagnosis. This novel molecular test requires the expression profiles of only 15 genes. Moreover, this is a functional test based on the analysis of senescence- and immortality-associated genes in tissue samples. The test was able to correctly predict 100% of cirrhosis cases. Twenty percent of HCCs displayed a borderline gene expression pattern, so that the classifier was not able to categorize them as HCC or cirrhosis. However, the test was able to predict the remaining HCC patients with 97.5% accuracy.

One of the hepatocellular immortality signature genes is ATAD2. By using techniques independent of microarray tools, we demonstrated *in vitro* that ATAD2 RNA and protein that are weakly present or not expressed in normal hepatocytes and fibroblasts are highly expressed in HCC cell lines. We also showed that ATAD2 protein levels go down in association with Adriamycin-induced senescence arrest in otherwise immortal Huh7 cells. ATAD2 protein is likely to be a chromatin modifier [48]. Its exact cellular function is unknown, but its overexpression in immortal cells, and in many cancer types [39], [68] is in favor of an essential role in tumor malignancy. Finally, our preliminary findings suggest that hepatocellular immortality signature genes such as ATAD2 may serve as promising HCC biomarkers.

## Supporting Information

Table S1 **Patient demographic data, diagnosis and disease etiology of clinical samples used for gene signature validation test.**
(XLSX)

Table S2 **Microarray gene expression data and literature review of 15 classifier genes.**
(XLSX)

Data S1 **Gene Set Enrichment Analysis (GSEA) results of gene expression data from Huh7 clones (immortal and senescent) and diseased liver tissues (cirrhosis and HCC).** “C2_All” curated gene sets of the MSig Database were analyzed. Commonly enriched gene sets in senescent/cirrhosis, immortal/cirrhosis, immortal/HCC, senescent/HCC groups are provided. The final worksheet summarizes 74 gene sets with significant co-enrichment (P<0.05) in both Huh7 clones and diseased liver tissues.
(XLSX)
